# Decoupling model descriptions from execution: a modular paradigm for extensible neurosimulation with EDEN

**DOI:** 10.3389/fninf.2025.1572782

**Published:** 2025-08-07

**Authors:** Sotirios Panagiotou, Rene Miedema, Dimitrios Soudris, Christos Strydis

**Affiliations:** ^1^Neuroscience Department, Neurocomputing Lab, Erasmus MC, Rotterdam, Netherlands; ^2^Microlab, School of Electrical & Computer Engineering, National Technical University of Athens, Athens, Greece; ^3^Quantum and Computer Engineering Department, Electrical Engineering, Mathematics and Computer Science Faculty, TU Delft, Delft, Netherlands

**Keywords:** computational neuroscience, spiking neural network, simulation, accelerated computing, high performance computing, NeuroML, software architecture, plugins

## Abstract

Computational-neuroscience simulators have traditionally been constrained by tightly coupled simulation engines and modeling languages, limiting their flexibility and scalability. Retrofitting these platforms to accommodate new backends is often costly, and sharing models across simulators remains cumbersome. This paper puts forward an alternative approach based on the EDEN neural simulator, which introduces a modular stack that decouples abstract model descriptions from execution. This architecture enhances flexibility and extensibility by enabling seamless integration of multiple backends, including hardware accelerators, without extensive reprogramming. Through the use of NeuroML, simulation developers can focus on high-performance execution, while model users benefit from improved portability without the need to implement custom simulation engines. Additionally, the proposed method for incorporating arbitrary simulation platforms—from model-optimized code kernels to custom hardware devices—as backends offers a more sustainable and adaptable framework for the computational-neuroscience community. The effectiveness of EDEN's approach is demonstrated by integrating two distinct backends: flexHH, an FPGA-based accelerator for extended Hodgkin-Huxley networks, and SpiNNaker, the well-known, neuromorphic platform for large-scale spiking neural networks. Experimental results show that EDEN integrates the different backends with minimal effort while maintaining competitive performance, reaffirming it as a robust, extensible platform that advances the design paradigm for neural simulators by achieving high generality, performance, and usability.

## Graphical Abstract

**Figure:**
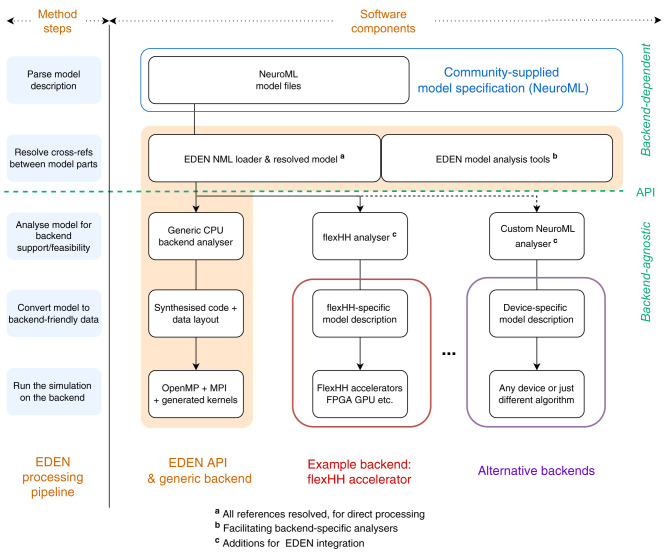
EDEN's multi-backend architecture. Software components included in the first EDEN release are shown with a colored background. The components marked with^c^ represent the necessary additions required to integrate arbitrary backends into EDEN.

## 1 Introduction

A rapidly growing subfield of computational biology is computational neuroscience, which is about running numerical models of natural (or nature-inspired) neural structures, so as to investigate the network dynamics that produce the marvelous capabilities of the nervous system. What makes computational neuroscience interesting from a *computer-science* standpoint is that the formulations for neurons and synapses is exceptionally diverse among models in use, to the degree that different processing algorithms, data schemes and numerical methods are needed for different models. To make matters worse, state-of-the-art modeling involves increasingly higher-model complexity, as the understanding of single-neuron dynamics becomes clearer, while the co-simulation of hybrid-complexity models is at times also necessary.

In this rapidly evolving and challenging landscape, neural-simulation design hinges predominantly on three, competing goals:

- **Generality** in supported model dynamics; that is, supporting more neuron types in conjunction, more dynamics equations, more interactions between parts of the model;
- **Computational performance**, so that more complex models can be explored at larger scales in space and time;
- **Usability**, so that users can express and implement the proposed models with as little effort as possible.

**Figure 1:**
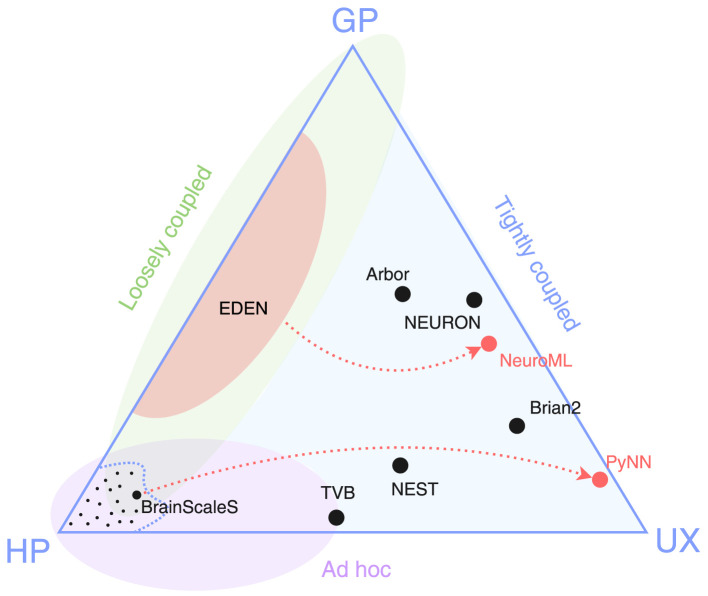
Simulator triangle. The three vertices represent competing design goals: general purpose (GP), high performance (HP), usability (UX). Dots in black represent existing simulator designs, and dots in red represent cross-simulator model specification languages. The area enclosed by the dashed curve, along with the small dots within it, represents the multitude of high-performance, *ad-hoc* solutions tailored to specific SNN models. The three shaded regions indicate the reach of three different approaches to simulator design: *ad hoc*, loosely or tightly coupled (introduced in Section 2).

We put forward the *simulator triangle* shown in Figure 1 as the taxonomical space of neural simulators built by the neuroscience community. Despite their large diversity and coverage, we propose that all of them fall short of at least one of these goals: General-purpose (or simply, general) and performant simulators involve implementing the whole model and simulation as a custom program; performance-aware programming (whether linear-algebra or tensor-based, or platform-specific coding) adds to the required effort, thus losing on usability. In contrast, highly performant and user-friendly solutions [e.g., TVB (Sanz Leon et al., 2013), CARLsim (Niedermeier et al., 2022)] focus on a narrow model type, thus have limited generality. Finally, simulators focusing both on generality and usability end up sacrificing processing speed in performing specific tasks for the sake of a streamlined, uniform interface (Hines and Carnevale, 1997).

Figure 1 suggests that there is a general lack of simulators able to deliver high simulation speeds without sacrificing their general applicability. After careful study of the field of domain-specific simulators, we identify two key design decisions that have prohibited notable solutions in that triangle niche. The problem lies in that each simulator (a) is **built around a single high-level simulation algorithm and data format** (henceforth collectively called *engine*) for processing the information; and (b) **accepts models expressed in a modeling language that is tightly bound** to the aforementioned internal algorithm.

These two decisions *constrain* greatly the formalism of models that a simulator can support through its internal design as well as for which model types or use cases, and for which computer systems it is an efficient design. At the same time, porting a model across simulators, that is, porting between the design-dictated formalisms takes considerable effort (Goddard et al., 2001; Davison et al., 2009; Gleeson et al., 2010). However, **models do not have to—in principle—be expressed in a system-specific language**. The ongoing dynamics can be described objectively in a simulator-agnostic format (as usually captured at the time of publication), such as natural language or mathematical language (e.g., ODEs); for instance, NeuroML v2 (Cannon et al., 2014) can capture many Spiking Neural Network (SNN) models. This observation is important since moving from a simulator-agnostic format to a simulator-dependent one is easy, yet it is much harder to “decompile” in the opposite direction. Hence, when there is a machine-readable, simulator-independent way to fully describe SNN models, we do not have to select a single simulator engine and, then, proceed to make its codebase cumbersome and less efficient by adding an ever-expanding list of features. We can instead express the intended model, and then deploy the engine that serves our needs best each time.

In response to the limitations outlined above, the *EDEN* simulator was proposed in Panagiotou et al. (2022), introducing two key design principles. First, rather than developing a new model-specification language, **EDEN adopts the existing** ***NeuroML*** **standard** as input, which is agnostic to the underlying simulation mechanisms. This choice is depicted as a dashed red arrow connecting EDEN to NeuroML in Figure 1. Second, **EDEN embraces a** ***loosely coupled*** **architecture that decouples the simulation engine from the rest of the system**. This allows different simulation backends to be flexibly integrated for different parts of a model, with the goal of improving performance or leveraging existing tools. Engines are thus reconceptualized as *backends* to a common model input *frontend*, rather than being the central component of the simulation system, as illustrated in Figure 1. The aforementioned design decisions permit EDEN to provide model generality and usability while, at the same time, achieving performance competitive with other simulators by generating code that it tailored to the model to be run each time. In its original form, EDEN was validated using a generic CPU-based simulation backend. In the present work, we extend its architecture to fully support arbitrarily different SNN simulation backends, including both software simulators and specialized hardware platforms. Although this extensibility was anticipated in the original design, it is only in this manuscript that we (a) introduce the necessary API extensions and (b) demonstrate the extended framework in practice using two fundamentally different backends.

In the following, we revisit EDEN's multi-engine framework and supporting infrastructure, and lay out in detail a methodology for attaching engines with disparate capabilities and target hardware, without sacrificing processing performance or redesigning the engines. We, then, proceed to validate this methodology by incorporating flexHH (Miedema et al., 2020), a competitive, exascale-ready, Field-Programmable Gate Array (FPGA)-based engine that simulates biophysically-detailed neuron models as an alternative to EDEN's universal engine, and evaluate EDEN's performance under different neural-network compositions for both engines. The goal here is to show that the EDEN framework offers (1) a short *time to solution* for incorporating custom simulation engines, (2) performance benefits, when they can be had, and (3) a clear, user-friendly methodology for other neuromodellers to replicate. In so doing, we aim to reaffirm the novel paradigm that EDEN brings about and to fulfill the three foundational goals expressed in the beginning of this section. Concisely, the contributions of this work are:

1. A taxonomy categorizing existing approaches to SNN-simulator design.
2. Further development of a novel paradigm for designing general, efficient, and user-friendly SNN simulators, exemplified by EDEN.
3. A systematic methodology for extending EDEN-based accelerated simulation backends, validated through the integration of two distinct, heterogeneous simulation platforms.
4. An experimental evaluation of the performance gains enabled by these backends, demonstrating the benefits of EDEN's multi-engine support.

The rest of the manuscript is organized as follows: In Section 2, we review related works and provide a taxonomy of current simulator designs, highlighting the different paradigm that EDEN brings about. In Section 3, we provide a quick overview of the EDEN architecture and its key properties essential to incorporating diverse, optimized backends. We, then, proceed to outline a methodology to do so which can be repeated for any alternative backend. In Section 4, we proceed to demonstrate how two disparate, preexisting simulator backends—flexHH, based on a FPGA platform, and SpiNNaker, based on an MPI CPU cluster—can be connected to EDEN. In Section 5, we evaluate the functionality and performance of EDEN's universal backend against the two example backends, and discuss our findings. In Section 6, topics worth future investigation are discussed. Paper conclusions are drawn in Section 7.

## 2 Related work

The simulator triangle of Figure 1 attempts to illustrate the underlying tradeoffs in neurosimulator design when trying to score high in all three aspects of generality of use, high simulation speed and user friendliness. The figure also shows another taxonomy of the various proposed simulators among so-called *ad-hoc, tightly coupled* and *loosely coupled* simulators. Due to various implementation or historical reasons, we estimate that *ad-hoc* simulators have mostly focused on high performance and, to some degree, user friendliness. Tightly coupled simulators have typically prioritized a broad coverage of supported neural models along with user friendliness, while attempting (but not always achieving) some measure of good simulation performance. Finally, our focus in this manuscript are loosely coupled simulators which attempt to cover the gap between high performance and generality of use, which is the toughest challenge to tackle.

**Figure 2:**
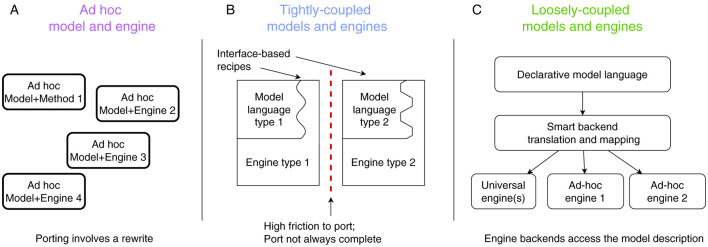
Different neurosimulator design approaches: **(A)** *Ad-hoc* solutions, made for specific experiments and offering scarce re-usability. **(B)** Tightly coupled simulators, designed around one simulation algorithm and recipes that express models as customisations of that algorithm. **(C)** Loosely-coupled simulators, which ingest platform-agnostic model descriptions and map them to best-suited (existing) simulation platforms.

Figure 2 presents a conceptual overview of this SNN-simulator taxonomy along with the design philosophy behind each one. As we will explain next, modern-day neurosimulators belong under classes (A) or (B), relying on a single simulation algorithm and tightly coupled, proprietary modeling languages, which constrain the range of models and use cases they can efficiently support. This design limits interoperability, making it difficult to port models between simulators and necessitating a shift toward simulator-agnostic, machine-readable formats for describing models; this is class (C).

### 2.1 *Ad-hoc* model implementations

The so-called *ad-hoc* way of implementing (simulations for) models (Figure 2A) forgoes support for multiple use cases and focuses on the needs of a particular experiment line. This exclusive focus on the immediate requirements allows for a perfect fit of the simulator design to the experiment, and thus is the only way to implement extreme-scale simulations, that exploit the available (e.g., neuromorphic) technology to the limit. Examples of this approach are FACETS (Schemmel et al., 2010), Nepteron (Beuler et al., 2017) or other custom simulators like (Yamaura et al., 2020).

Unfortunately, this engine-design approach incurs high costs in time and effort, since everything needed for the simulation is built from scratch. If, while exploring the model, it is desirable to modify or extend it, the necessary changes may come in conflict with key assumptions of the design at the technical level. In that case, the necessary significant modifications may escalate to a redesign of the whole simulation system. And when an experiment is completed, there are few re-usable parts for follow-up projects; hence most of the setup cost is recurring for each experiment.

### 2.2 Tightly coupled simulators

A different way to build a neural-simulation system (Figure 2B) is to start with a specific engine—which is effective for a certain sub-class of neural models—and expose specific “code hooks” via which the engine can be generalized (such as the post-synaptic current trace of a synapse type, or the dynamics of an ion channel population). This produces a generalized (but *not* truly general) simulator, that can be used to implement many new, neural models within the originally intended class. Each targeted model must then be expressed as an *engine-specific* “recipe” that modifies the simulator hooks, so that running the simulator will produce the intended behavior. Examples of this approach are NEURON (Hines and Carnevale, 1997), Brian2 (Stimberg et al., 2019), NEST (Gewaltig and Diesmann, 2007), and Arbor (Akar et al., 2019); further examples can be found in Blundell et al. (2018). The recipe constructs the neural network to be simulated and directly controls the simulation's progress through an experiment. This approach significantly enhances productivity for modelers compared to *ad-hoc* model implementations. Modifying the model under investigation is straightforward by adjusting the recipe at the engine's code hooks. However, this monolithic “recipe-in-engine” methodology introduces challenges for both simulator users and developers, in terms of modeling ease as well as simulation performance. Modeling-wise, the challenges are as follows:

- **Limited model portability:** since different simulators rely on distinct engines with varying formulations and code hooks, converting a recipe created for one simulator for use in another can be a *labor-intensive* task. This is especially problematic because the boundaries between model classes are often fluid, and neuroscience practice frequently involves combining features from different classes (e.g., integrating biophysically detailed neurons into a point-neuron network, or vice versa). This “hybridisation” of models may lead to another complication: if a model spans multiple classes extensively, no single engine may fully support it. In such cases, modelers are compelled to adopt *ad-hoc* solutions, such as exchanging information between simulators that handle different parts of the model or constructing a complete, bespoke simulator tailored to the specific model.
- **High model-engine dependence:** parts of how the engine works are implicitly included to the functional behavior of the model, which complicates understanding and unambiguously describing models that are written as recipes.

Performance-wise, tightly coupled simulators incur additional penalties:

- **Limited performance portability:** simulator engines are—by definition—obsolete the very minute they are designed. The reason is that each engine is optimized for a contemporary computing platform. As technology evolves, the assumptions that led to efficient execution on the original platform may not hold when migrating to a new one.
- **Limited performance optimisations:** related to the previous problem, any code hooks that an engine provides are—by design—tailored to said engine. When the engine is modified to exploit new improvements in technology, those hooks—meant for generality—may prevent effecting necessary engine optimisations.

### 2.3 Loosely coupled simulators

The aforementioned limitations of the so-called tightly coupled simulators gave rise to the so-called loosely coupled simulator designs; see Figure 2C. Although we are not the first to propose solutions in this direction (see the discussion on PyNN by Davison et al., 2009, below), we are the first to attempt this taxonomy and clear separation among design approaches, as shown in Figure 2.

In this approach, models are described in a *declarative, unambiguous, engine-agnostic* way and can be simulated using different engines. One such modeling language is NeuroML (Goddard et al., 2001; Gleeson et al., 2010; Cannon et al., 2014; Sinha et al., 2024) which captures a model closer to its published textual form and the original intent, catalyzing model adoption and exchange. These same properties also permit a “pure” mathematical inspection of a model, without being polluted by engine-induced (and in a sense, unnecessary) model behavior.

This approach trades some additional development effort due to decoupling,^1^ for model-description reuse and the ability to switch engines at will. Reusable descriptions are beneficial to the research community and, at the same time, interchangeable engines allow employing best-in-class alternatives each time. Loosely coupled simulators, thus, bypass the modeling issues of model expressiveness and *model-engine dependence* by excluding any engine-related assumptions from the models. Dependence on a particular engine to fill in parts of the behavior is also avoided for the same reasons. Lacking any engine-incurred assumptions, *model portability* is ensured since any part of the model may be described in the most semantically appropriate formalism. In terms of *performance portability* and potential *optimisations*, this approach is only constrained by inherent model idiosyncrasies (if any) that may or may not map efficiently onto a given platform.

#### 2.3.1 The EDEN simulator

Recently, a new simulator for spiking neural networks, EDEN (Panagiotou et al., 2022) was developed. A core design choice in EDEN was to *not* develop a new model-design environment and specification language for the simulator, but to instead directly load and run model files expressed in NeuroMLv2 (Cannon et al., 2014). The aim of EDEN is to run a *production-ready* range of models^2^ with high computational performance, without forcing users to program against the engine but, instead, by directly reading models in an unambiguous format such as NeuroML; see dashed, red line connecting EDEN to NeuroML in Figure 1. All in all, the major novelty of EDEN is that it decouples:

- the abstract description of the mode at the top; and
- the code and data representation used to simulate the model.

Computational performance is achieved through, first, analyzing the various model parts (namely, neurons, synapses and experimental setup) and, then, translating them to suitable machine code. The type of generated code depends on the per-case available simulation platforms; for instance, just-in-time code generation takes place for standard CPUs, while custom (3rd-party) code can be leveraged for GPUs, FPGAs, AI chips, and so on.

#### 2.3.2 Other multi-backend approaches

Perhaps the most well-known, all-around alternative approach to EDEN is PyNN (Davison et al., 2009). PyNN is a Python interpreter-based language for constructing SNN models and simulating them, which delegates the simulation workload to different simulators through a uniform interface, provided that they support the described model features. PyNN originated in the FACETS project (Schemmel et al., 2010)—the predecessor of BrainScaleS (Pehle et al., 2022)—stemming from the pragmatic need to run portable experiments on various simulation platforms (both software- and hardware-based); see dashed, red line from BrainScaleS to PyNN in Figure 1.

As discussed above, Brian2 (Alevi et al., 2022) is a tightly coupled simulator since its event-handling order^3^ is implicitly defined into the engine rather than explicitly defined in the model. While the later-added GeNN backend (Stimberg et al., 2019) offers some degree of decoupling, its differing event-handling order (in all but simple models) can result in inconsistent execution compared to Brian2. Thus, Brian2 falls short in that it does not provide consistency guarantees. It is because of this issue that it is not classified as a proper loosely coupled simulator.

The distinction between these prior developments and the EDEN architecture lies in two key aspects: (a) we introduce a methodology that enables a repeatable approach for integrating different simulators with alternative simulation engines, and (b) by leveraging the open specification format of NeuroML, our architecture remains independent of any single simulation environment's formalism and its associated assumptions.

## 3 Methods

### 3.1 The EDEN architecture so far

SNN models and simulation engines are in constant flux over time, making it difficult for a single simulator to handle the plethora of targeted experiments. This observation led to the development of EDEN, which leverages best-in-class simulation backends at each point in time. SNN-model inspection takes place so as to maximize simulation performance given available computing resources. As already mentioned, EDEN adopts NeuroML for SNN modeling, making no assumptions about the simulation backend.

The first release of EDEN (Panagiotou et al., 2022) had implemented a single, *generic*, simulation backend, targeting general-purpose CPUs. Under this design, parts of the code template are adapted independently for the different parts of the neural network to be simulated, but certain high-level design decisions remain fixed. This generic backend is, however, not the sole target of development. In line with the EDEN architecture which prescribes specialized engines and, thereby, backends, this existing backend is intended to complement more optimized future designs, as an always-applicable, last-resort option. Generic backends can, hence, guarantee a *lower bound* on the computational performance that EDEN can offer. In this section, we will present the EDEN methodology for replacing or adding arbitrary, alternative simulation backends.

### 3.2 The general engine-integration process

The EDEN processing pipeline, originally discussed in (Panagiotou et al., 2022, figure 2), is shown in Graphical Abstract (left: method steps) and consists of a sequence of stages between the model files and the code and data to run the simulation:

1. Parse the NeuroML files that describe the model.
2. Resolve cross-references between the model's entities, to aid analysis.
3. Analyse the model's parts before deciding how to implement them.
4. Convert the model into a configuration (code and data) for simulating.
5. Execute the generated code on the model data.

The last three stages are called the “backend” of the processing pipeline and, so far, they have been implemented in the context of the generic, CPU backend described above. This backend can handle all parts of a NeuroML model and it adapts the simulation code and data structures separately for each part, using the leaf-level “signatures” of what has to be simulated (Panagiotou et al., 2022, sec. 2.4.2).

This same pipeline can also be employed with different simulation backends (either software- or hardware-based). In such cases, the last three stages are adjusted as follows:

1. Analyse the model for optimal mapping onto the specific backend.
2. Convert the model into a backend-specific configuration for simulating.
3. Run backend simulation with the specified configuration.

In **Graphical Abstract**, the original EDEN components are highlighted in orange color, indicating the extensions done for this work. As the figure reveals, the EDEN architecture entails a backend-agnostic component and necessary, backend-specific components (blocks with colored outline). These components need to be authored every time a new simulation backend is hooked up to EDEN. The interface between these backend-agnostic and backend-specific components is called the EDEN API, to be discussed next.

### 3.3 An overview of EDEN's API

Since NeuroML assumes no particular simulation engine, EDEN delegates to each backend how to inspect and implement the user-specified SNN model. The main point of contact between EDEN's backend-independent part and the various backends is therefore exposing the model to the backends. In fact, this is the only strictly necessary part of the interface; the API also offers some auxiliary tools which do not provide additional information but may assist backends and their analysers to interpret the NeuroML model.

#### 3.3.1 Model interface

**Figure 3:**
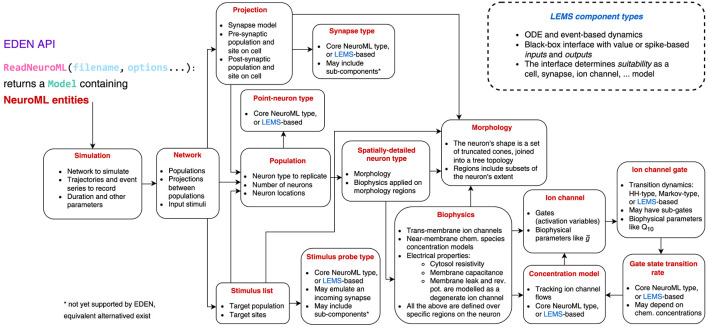
EDEN's minimal API laying out the structure of NeuroML simulations.

The main part of the EDEN API is the ReadNeuroML function which, given the file location of a NeuroML model file (and other NeuroML files that it probably includes), returns the Model data structure that represents the parsed and cross-referenced NeuroML model. The Model comprises the set of NeuroML entities described in the input file(s), including the references between entities. Details about and the reference relationships between different types of NeuroML entities are shown in Figure 3. An important purpose of resolving cross-references is that, at this point, the model information is not simply parsed but also *semantically validated*; if the provided model is successfully processed, it is *guaranteed* to be consistent and implementable by a compatible backend. One could, as well, load the NML with a custom new parser, but in that case they would also have to validate the semantic information implicit in NeuroML: that is, validate and resolve cross-references between NeuroML entities, and ensure that biophysical mechanisms are defined and are compatible with their uses in the model description. EDEN offers the aforementioned routine in the API that takes care of these considerations (for the cases that a more specialized parser implementation is not needed).

#### 3.3.2 Optional API

More than providing an actionable model description, EDEN's API also offers additional, optional tools. These auxiliary parts of the API can assist common tasks with (1) *model-related analysis*, for example, in determining compatibility between neuron models and the synapse models attached to them, mapping the geometry-based, neuron-site designators to specific compartments, and unit conversion; (2) “*signature”-composition facilities* for analyzing and processing LEMS components, and generation of C-like simulation code (of interest to backends that can work with generated code); and (3) *basic input/output functions* that follow the NeuroML convention for simulation-data formatting, which can be replaced with higher-performance implementations as needed.

Keep in mind that, as far as the EDEN architecture is concerned, the presented interface can be implemented in any formalism (for example, a text-based intermediate-representation language). We have implemented it as C++ headers and data structures in view of the existing needs for (a) a streamlined integration process (i.e., no need for learning and writing a parser for yet another data format vs. walking through structs), and (b) high-performance data processing (also at initialization time), which is necessary for large-scale models.

#### 3.3.3 Backend design guidelines

Although the specific methods depend intimately on the internal design of each backend, the analyser component for each backend must invariably perform the following tasks to run a user-provided simulation:

1. Explore the model's structure by traversing the references between the NeuroML entities involved.
2. Given the interactions between model parts, determine whether and how each can be simulated by the backend.
3. Transform the given NeuroML model to a backend configuration that simulates the model.

One path to developing a new simulation backend is to cover the *full set of models* expressible in NeuroML. Naturally, covering so wide a range comes with certain complexities in simulating all types of model parts and interactions thereof, and on top of that supporting custom-dynamics models (described through LEMS) for each of these entities. This complexity is inherent to simulating diverse SNN model types, whether using the EDEN framework or not. It follows that the model analyser for such a backend will also involve a certain level of complexity, just to handle the full set of NeuroML entities.

Another approach is to assume a *very narrow range of supported models*, and take advantage of this narrow scope to develop a backend with a highly specialized engine. This case is, in fact, close to the “*ad-hoc*” design approach presented in Section 2.1. In this case, the analyser's job is to directly map the model description provided by the API to the corresponding engine's features and customisable parameters, and reject any other (e.g., neuron, synapse) model part that this specialized backend cannot simulate.

## 4 Implementation and experimental plan

**Figure 4:**
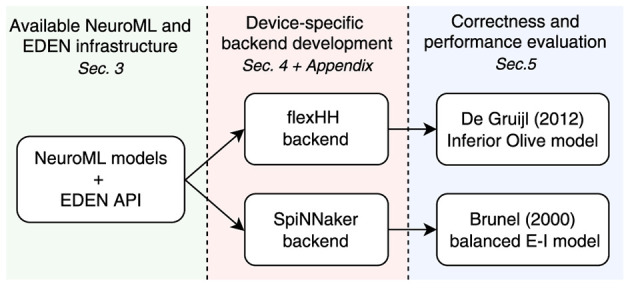
The backend implementation and evaluation plan.

One of the central points of this paper is that developing a backend-specific *analyser* involves two tasks: work to process the NML hierarchy and work to configure the backend's engine, both key to maintaining a loosely coupled simulator architecture. In this section and Appendix A, we will demonstrate how this same process is applied for completely different simulation backends, within the model limitations of each. These demonstrations are purposefully detailed so as to enable any interested researcher to replicate the process for any other potential backend; details non-essential for a first reading have been moved to the Appendix.^4^ We thus validate our EDEN engine-integration methodology as follows (see also Figure 4):

- Select two accelerated simulation platforms with disparate feature sets (flexHH and SpiNNaker);
- Use the EDEN API to create backends targeting these platforms, mapping NeuroML features to platform-specific configurations as necessary;
- Validate the integrated backends and evaluate simulation correctness, as well as simulation speed, compared to EDEN's existing generic backend.

### 4.1 Backend case 1: the FPGA-based flexHH simulator

flexHH (Miedema et al., 2020) is a FPGA-based design for simulating SNNs of the standard Hodgkin-Huxley (HH) model and other HH extensions including multicompartmental cells and gap junctions, while delivering very high simulation speeds. These speeds are achieved by following the *dataflow* processing paradigm, which forgoes random access of data for pre-determined sequential access and deep overlapping of calculations, so that the data “flows” from one calculation to the next. However, this dataflow design imposes certain limitations on the neuron models that can be simulated, as described in a next section. The interested reader may refer to the paper for the full details of the accelerator's design and modeling scheme. Here, we will focus on the key design decisions that impact the feasible range of models and on the flexHH analyser.

#### 4.1.1 flexHH architecture and model representation

The flexHH design consists of a dataflow algorithm that advances the state of neurons for each time step of the simulation, and the model's data arranged in a layout amenable to the dataflow algorithm. The algorithm has been designed ahead of time and does not change frequently; modifications for, e.g., different gap-junction dynamics have to be designed up-front as alternative *FPGA configurations*. Run-time decisions about the calculations to be done are encoded and included in the model data. The data layout is assembled as three sequences of “per-cell”, “per-compartment,” and “per-gate” data for the whole network, and one dense matrix containing the synaptic-connectivity weights.

**Figure 5:**
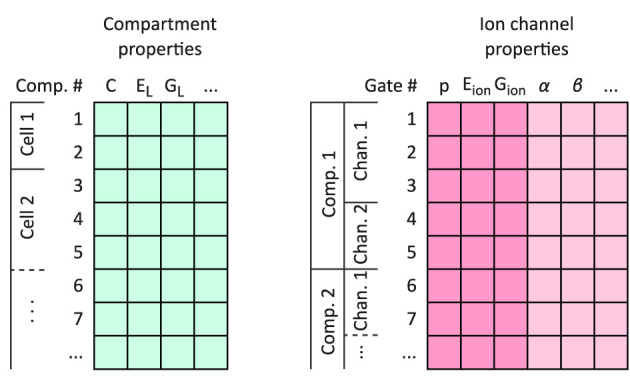
The flexHH memory layout for neuron models. Neuron data are stored using two arrays of records: one for per-compartment information, and on for per-ion-channel and per-gate data. All compartment entries for a neuron are laid out sequentially, immediately followed by the entries for the next modeled neuron. The mapping between neurons and their compartment ranges, and compartments and the ion channels that they contain, are tracked by integer “start-index” vectors per-neuron and per-compartment. Individual ion channels are demarcated on the gate-data array by a flag on the last gate entry per ion channel; per-channel data on the gate entries are only valid for these last entries. Per-channel information on the gate-data entries are highlighted with a deeper color.

It is important to keep in mind for the following that compartments, channels and gates are laid out in a *nested sequential* manner; all compartments of a cell, all channels of a compartment, and all gates of a channel are listed and considered in a certain order, to aid data-flow processing (see Figure 5).

#### 4.1.2 Models as supported by the accelerator

Each *neuron* in the model comprises one or more compartments, which are electrically coupled to each other by ohmic resistors. Compartments must be connected in a *linear-chain* topology. The compartment's physiological parameters are the electrical capacitance, passive leak conductance and membrane reversal potential, axial conductance to the preceding compartment in the neuron (when applicable), and a (direct) current-clamp stimulus with programmable start time, duration, and amplitude (set to zero when not present). Each compartment may contain a number of ion channels, and an ion pool that some of the ion channels contribute to.

*Ion channels* follow the HH formalism (Hodgkin and Huxley, 1952) of independent gate populations with one open and one closed state, so that the “gate variable” is the fraction of each gate population that is in the “open” state. The momentary transition dynamics between states is described as α, β that are the rates of transition from open to closed and closed to open respectively, or *ss*, τ for the steady-state value of the gate variable and the time constant to converge to by. Each of these expressions can be programmed as one of four different formulae of univariate functions, each with arbitrary coefficients. The argument to these rate functions can be either membrane voltage, or the state variable of the previous generalized “gate variable” in the sequence (this can be the ion concentration to model ion concentration-activated ion channels, as explained in Section A.1.1.1).

As *synaptic connectivity* is the most computationally demanding part of the model for large network sizes, it also the most specialized function in this accelerator. Neurons are assumed to be coupled via gap junctions only, which connect the first compartments of each neuron pair. The voltage difference-current relationship is an anti-symmetric function, that is fixed per FPGA configuration and only a different coupling strength can be specified for each pair of neurons. These design decisions about synaptic connectivity appear to be the most limiting aspect of the accelerator with regard to reuse.

Further technical details about this accelerator and interfacing with it can be found in Appendix A.1.

### 4.2 Backend case 2: the SpiNNaker simulator

#### 4.2.1 The SpiNNaker architecture

SpiNNaker (Furber and Bogdan, 2020) is a large computer cluster tailored to the requirements of SNN simulation. Instead of the typical large off-the-shelf server computers running a general-purpose operating system, this cluster's nodes are multi-core systems-on-chip (SoC) running bare-metal application code (and some minimal platform-control firmware), to allow for more parallelism in total. Each node has sixteen CPU cores available for applications; each core is designed to simulate up to a thousand neurons; each circuit board houses forty-eight nodes communicating in a hexagonal grid topology (also implemented across circuit boards); a system can have more than a thousand boards, for simulating up to (around) a billion neurons. Intercommunication between nodes (and typically cores) happens only as tiny (up to 32 bits of payload) datagrams meant to represent individual neuron firings, favoring low latency over bulk throughput and reliability. Occasional datagram loss is acceptable assuming that it does not disrupt the network's overall behavior.

This system can be either programmed directly (to be briefly discussed later) or set to run SNN simulations described through the PyNN language. The latter option is recommended since it offers fast development time, a lowered technical barrier to access, and an already platform-optimized engine for simulating SNNs.

#### 4.2.2 The PyNN description language and its limitations/constraints

As mentioned in Section 2.3.2, PyNN allows for expressing a limited subset of SNNs:

1. It covers only point-neuron models, i.e. those where the neuron's morphology is simplified away;
2. It considers only the “classic” model of chemical synapses with strictly post-synaptic effects; hence the synapses are modeled as entities attached to post-synaptic neurons.
3. Furthermore, the only post-synaptic potential (PSP) models allowed are those where multiple incoming potentials can be linearly summed (Lytton, 1996) into a *cell-specific* set of state variables, such as impulses, exponentially-decaying current or conductance, and generally constant-coefficient Markov kinetics. This is, however, only possible when the dynamics equations are exactly the same for all such synapses, i.e. share the same dynamical parameters (such as time constants).
4. Each PyNN neuron model is hence bound to a certain PSP model, allowing one set of PSP parameters for all excitatory synapses and another for all inhibitory synapses.
5. There is a very limited, predetermined set of neuron models (each tied to a specific PSP model as well) and synaptic plasticity models available to users.

Further technical details about this accelerator and interfacing with it can be found in Appendix A.2.

## 5 Results

In this section, we report our findings based on simulations conducted using the two EDEN-connected backends: flexHH and SpiNNaker. For each backend, our presentation entails two aspects: (a) functional validation, and (b) performance evaluation, both with respect to the native, CPU-based, EDEN backend.

### 5.1 Experimental setup

We demonstrate connecting two distinctly different backends to EDEN so as to showcase the versatility of our approach but also to highlight inherent benefits and limitations of each one of these backends. While flexHH is a FPGA-based, hardware backend targeting biophysically detailed, multicompartmental SNNs, SpiNNaker is a popular distributed-computing cluster based on ARM CPUs and natively supporting artificial SNNs (e.g., with integrate-and-fire neurons). Table 1 summarizes the technical specifications of each platform.

**Table 1:** Technical specifications for the three SNN simulation platforms used in this work.

**Specification**	**EDEN generic**	**flexHH**	**SpiNNaker**
	**(CPU)**	**(FPGA)**	**(CPU cluster)**
Device model	AMD 2,950X	Xilinx XCVU9P	Custom ASIC
Architecture	×86–64	Fixed-function	ARMv5
Circuit board	Gigabyte Aorus X399	Maia DFE	Custom interconnect
#nodes/cores	1 module × 16 cores	1 module	57,600 nodes × 16 cores
Compute throughput [Gop/s]^†^	896	87.2 (actual)	184,320 (6.4 used)
DRAM throughput [GB/s]	93.9	45	0.9 per node
L1 Cache [B]	64k I$ per core	75.9M (BRAM)	32k I$ per core
	32k D$ per core		64k D$ per core
Base clock frequency [GHz]	3.5	0.17	0.2
Power consumption (TDP) [W]	180	23 (actual)	100k (2 used)
Semiconductor process node [nm]	12	16	130

All reported CPU figures are theoretical peak numbers and that a small part of the SpiNNaker platform was used for the validation and performance benchmarks.

^†^Operations are mentioned here to account for the fact that SpiNNaker only supports integer arithmetic. For the remaining cases, operations equal single FLOP.

### 5.2 flexHH evaluation

**Figure 6:**
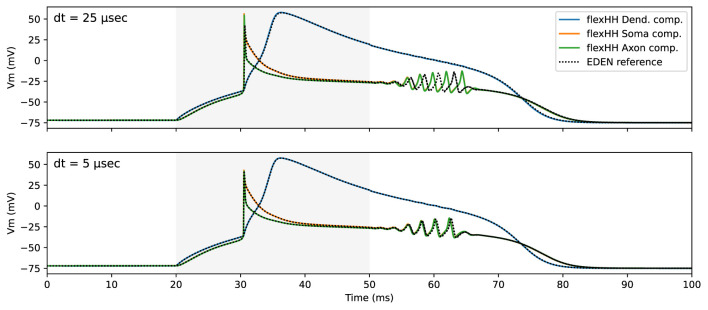
flexHH functional validation against EDEN generic CPU backend for a small **(bottom)** and a larger **(top)** sim-step size. The time interval during which current stimulus was applied is shaded in light gray.

To validate the model-mapping process when deploying the flexHH backend, we constructed a neuron model in NeuroML that is mathematically equivalent to the simplified three-compartment model of Inferior-Olivary(IO) neurons of De Gruijl et al. (2012). This model was run on flexHH in previous work (Miedema et al., 2020), and it utilizes all neuron-modeling features supported by flexHH. Figure 6 captures the flexHH-backend **functional validation**; it shows the simulated-neuron's electrical activity when the “dendrite” compartment is subjected to a current-pulse stimulus, as simulated by flexHH and (for comparison) the generic CPU backend. It is interesting to note that—upon closer inspection—the generic EDEN backend shows *worse* accuracy. This is due to the undershoot inherent in *backward Euler* used by EDEN,^5^ compounded by current integration options in the generic backend that have backfired in this case.

To perform a fair **performance evaluation**, we constructed and ran neural network models within the (more restrictive) design space that flexHH permits. For this space, only electrical synapses (i.e., gap junctions) were supported, with the focus being on high-density graded connectivity which is typical in the inferior olive and, also, the most computationally intensive part of the simulation. We, thus, explored networks of IO neurons connected as Gilbert *G*(*n, p*) random graphs (Gilbert, 1959) for a varying number of neurons *n* in an uniform population, and probability *p* that any distinct pair of neurons may be connected via a gap junction.

Having constructed various network compositions (of *n* and *p*), we evaluated the achievable simulation speed for each of them when using either the EDEN generic CPU backend or flexHH one. The wall-time measurements were largely consistent between trials; this was expected due to the simulation methods' regular processing pattern that repeats for every fixed time step.Each simulation was ran for 1,000 time steps (the model was originally run with a time step of 50 μs, hence the simulated time is 50 ms and it would take 20,000 steps to simulate one second). Since the original flexHH engine runs *unmodified* at simulation time by design, any computational performance differences are expected only due to the initialization phase. There is indeed some overhead arising from using the flexHH backend—specifically, for parsing the NeuroML description (via EDEN's frontend) and converting it into device-native data structures. Initialization times are but a fraction of overall execution times, thus here we only report pure simulation runtimes.

**Figure 7:**
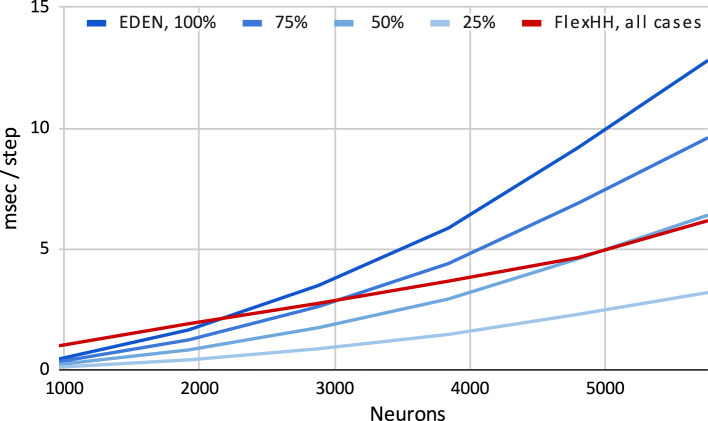
Execution time per simulation step for simulating increasing sizes and densities of the Inferior-Olivary nucleus network. flexHH favors simulations instantiating larger network sizes and/or higher synaptic connectivities.

Figure 7 shows time per simulation step for flexHH and the standard CPU backend when increasing both network population size and intraneuron connectivity density. Due to its design, flexHH always has the same execution time for a population size, regardless of how many connections are present, whereas the generic backend saves time by considering only the existing connections. As the figure reveals, flexHH becomes beneficial with increasing network sizes of higher connectivity density, which is exactly the purpose for which flexHH was originally designed and results agree with those of the original publication (Miedema et al., 2020). It is also interesting to notice the inflection points where flexHH outperforms the CPU and vice versa. It would be interesting to take a step further and imbue EDEN with the ability to choose the optimal simulation backend based on the (expected) performance behavior of each considered backend. This is hinted to in passing in Figure 2C (“*Smart backend translation and mapping” block*) but is a topic for a different discussion.

**Figure 8:**
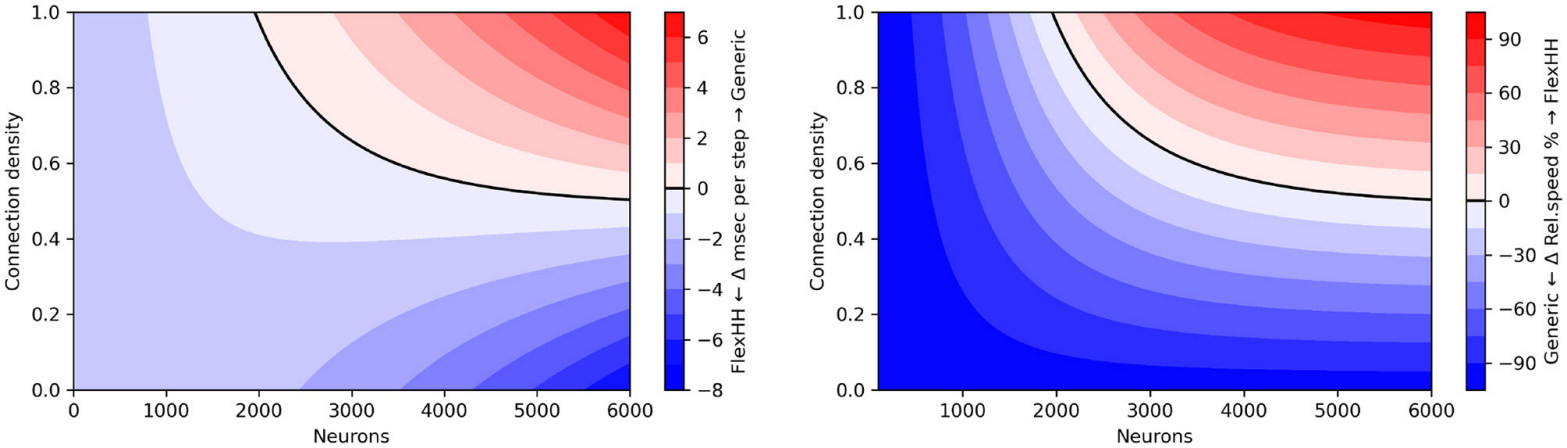
“Heatmap” performance comparison between flexHH and generic EDEN backend for varying population sizes and network densities. **(Left)** Run time difference per simulation step, in absolute terms. **(Right)** Speed percentage difference (in steps per second)

Figure 8 shows the difference of the two backends as a function of network size and connectivity density, when measured in terms of absolute runtime per simulation step and of speed percentage difference in steps per second. We observe that flexHH can deliver up to twice the speed for the more demanding cases. However, it is outperformed by the generic backend on a workstation-grade CPU, either (a) when the population size is around 2,000 or less, or (b) when *n* is lower than around 50%, and (c) in a small area near those two cut-off points.

Note that flexHH is capable of handling much larger network sizes (reportedly up to 18,240 neurons on a single FPGA card), with full connectivity. We could not evaluate for fully connected networks of more than 5,760 neurons using the EDEN backend due to a memory limitation that we encountered. In the current EDEN frontend design, all synapses are parsed from a NeuroML file, and the connection information is decoded into numbers for (among other things) the neuron population in the model, specific neuron in said population, specific neurite segment and distance along it—to designate both the pre-synaptic and the post-synaptic site. In practice, the thus parsed and decoded representation of a synapse adds up to 300 bytes per decoded  < connection> XML tag (on a 64-bit platform) and 100 bytes of decoded pre- and post-synaptic sites. Ultimately, this amounts to tens of gigabytes of working memory for 5,000 or more fully connected neurons, even if the eventual representation is much more compact (currently, 24 bytes for gap junctions of this type, for the universal backend). This issue can be fully resolved in follow-up work by (a) using the newly-formalized, memory-efficient HDF5 encoding for NeuroML v2 networks; and (b) interleaving connectome loading and backend processing in time via the “visitor” software design pattern (Gamma et al., 1994, p. 331).^6^

### 5.3 SpiNNaker evaluation

**Figure 9:**
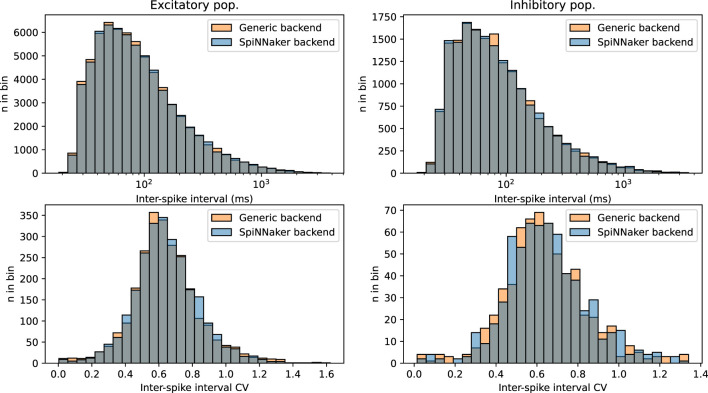
Overlapping histograms of neural activity statistics, for the SpiNNaker and generic EDEN backend, for the CUBA network with *n* = 4, 000 and *p* = 0.02. **(Top row)** Inter-spike intervals over all neurons; bottom row: coefficient of variation over each neuron's inter-spike intervals; **(left column)** excitatory population; **(right column)** inhibitory population. Compare with figure 24 of Brette et al. (2007). Note that the excitatory population is four times as large as the inhibitory population, and displays more predictable statistics as expected by the law of large numbers.

To validate the functionality the SpiNNaker backend and assess the computational merits of the simulation platform, we deployed the CUBA benchmark network from Brette et al. (2007) (originally published in Brunel, 2000), expressed in NeuroML. This is a rather simple network of neurons following the linear leaky integrate-and-fire (LIF) neuron model, split into two populations, one excitatory and one inhibitory; every neuron present is connected to (and hence influences) a randomly-selected subset of both populations, hence this model is also an instance of a directed Gilbert random graph with a *n* = 4, 000 and *p* = 2%. Post-synaptic interaction is modeled as an influx of exponentially-decaying current. The dynamical parameters of neurons and synapses are set to sustain a steady, yet chaotic firing activity. Figure 9 shows firing statistics for the two populations, when simulated with either the SpiNNaker or generic EDEN backend. The histograms for inter-spike intervals (ISI) and per-neuron ISI coefficient of variation (CV) closely match each other, and also the histograms originally shown in the aforementioned publication for the NEURON and NEST simulators. Note that neurons are firing asynchronously, in which case ISI and ISI CV are i.i.d. as explained in Appendix A.1 of the original model (Brunel, 2000). A two-sample Kolmogorov–Smirnov test for the data in each histogram does not indicate within a confidence interval of 10% that the underlying distributions are different for the two simulation platforms (*p*>0.1).

Following validation of the backend, we evaluated computational performance for varying size and density parameters of the CUBA network. The SpiNNaker platform is designed as a real-time system; each simulation step takes exactly one millisecond of real time, regardless of the size, connection density or activity of the simulated network. We can thus expect that workload-adaptive simulation engines (like most popular SNN simulators) can run small enough networks faster than SpiNNaker, and that accurate simulation on SpiNNaker is constrained by the platform's ability to handle each simulation step within 1 ms. Hence, SpiNNaker is best suited for large SNN models, and simulation speeds not much removed from real-time in either direction. The CUBA model is simulated with a time step of 0.1 ms; hence SpiNNaker runs it at 1/10 of real-time speed.

**Figure 10:**
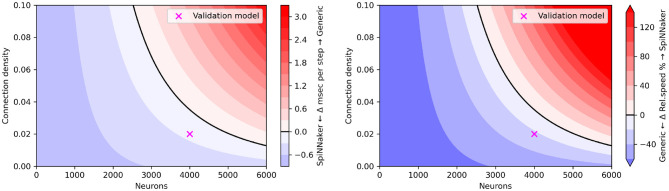
“Heatmap” performance comparison between SpiNNaker and generic EDEN backend for varying population sizes and network densities. **(Left)** Run time difference per simulation step, in absolute terms. **(Right)** Simulation speed difference, in relative terms. The original CUBA network configuration (which was used as the validation benchmark), is indicated with a purple cross.

Figure 10 shows the relative simulation speed for varying population size (up to 6,000 neurons) and density (up to 10%) parameters of the CUBA network. We observe that similar to the flexHH evaluation, the accelerator provides increased performance when the simulated network is both large (high neuron count) and densely connected; a difference with flexHH is that SpiNNaker runs at a *fixed* simulation speed regardless of the number of neurons. Note that there is necessarily a limit to how complex a model SpiNNaker can run, given its fixed time budget per step; but this limit exists far from the tested configurations, where the generic backend on a workstation-grade CPU is remotely competitive.

It is clear that EDEN delivers comparable results to SpiNNaker for rather small networks, as can be seen from Figure 10. This is simply due to the fact that, currently, EDEN's generic backend evaluates synaptic current for every synapse individually, as it must in the general case of parameter variability. One of the planned performance improvements for EDEN is to aggregate identical linear synapses as in Lytton (1996), which is expected to speed simulations up by at least an order of magnitude for sufficiently dense, point-neuron networks.

## 6 Discussion

The previous sections introduced current challenges in and compared the existing high-level approaches to simulator design, proposed a modular approach that allows on-demand deployment of different “back-end” simulation platforms, and demonstrated how existing highly specialized platforms can be straightforwardly integrated and exploited. As seen in the Section 5, performance gains from using one backend over others are conditional to properties of the simulated model, and extreme-scale models can call for performance improvements in the EDEN API itself. Technically inclined readers and designers of simulation engines can repeat the steps shown in this work for new backends and analysis tools, with the help of the EDEN user guide (https://docs.eden-simulator.org/, section “Extending EDEN”). However, the presented architecture also sets the stage for further development steps, discussed next.

### 6.1 Single- vs. heterogeneous-backend deployment

Currently, backend integration is implemented in a minimal form: the new backend operates either exclusively or in conjunction with the standard backend. However, this setup prevents specialized backends from being utilized when small, unsupported additions are introduced. An advanced alternative could involve heterogeneous deployment, where different parts of the network are assigned to different backends that communicate with one another. While this idea has potential, its practical success depends on several factors:

- The degree and immediacy of communication required between network components.
- The predominantly homogeneous coarse structures of most models, despite their fine-grained diversity.
- The limited spatial basis of connectomes, which constrains the utility of heterogeneous deployment.

### 6.2 Automatic backend selection

Another key observation we made was that, in both single-backend and heterogeneous cases, backend selection currently relies on user input. A promising direction for future work would be the use of the EDEN API to extract model analytics. This information could drive heuristics or machine-learning techniques to automate backend selection and optimize the model-deployment plan for performance. By reducing the burden on users and improving backend assignment, such automation could further enhance the usability and efficiency of the EDEN framework. This approach is akin to the well-known *optimal resource-allocation problem*. Inspiration can be drawn from modern-day, AI-fueled solutions such as Betting J.-H. et al. (2023) and Betting J. et al. (2023).

## 7 Conclusions

Simulation platforms in computational neuroscience have historically been constrained by tightly coupled simulation engines and modeling languages, which impose significant limitations on flexibility and scalability. Redesigning these platforms at a later time becomes costly and sharing models across different simulators is often cumbersome. Based on the EDEN neurosimulator, this paper proposes a solution through an approach that introduces a modular stack where abstract model descriptions are separated from execution. This design enhances both flexibility and extensibility in simulation platforms. The EDEN approach enables multiple backends, including hardware accelerators, to be integrated seamlessly without requiring extensive reprogramming. This ensures that switching between backends is straightforward and does not necessitate rewriting the entire simulation program. By utilizing NeuroML, engine or accelerator developers can focus on high-performance execution, while NeuroML users benefit from these enhancements without the need to develop simulation engines themselves. Additionally, the proposed method for incorporating arbitrary backends—that may utilize custom hardware along with generated code—provides a more sustainable approach for the computational-neuroscience community. This technique allows for easy integration of new backends, utilization of specialized models, and fallback to NeuroML and code-generation facilities when specific backends are unavailable.

The benefits of the EDEN approach are demonstrated via attaching two rather disparate backends: flexHH, a FPGA-based hardware accelerator for extended Hodgkin-Huxley networks of neurons and the well-known SpiNNaker platform, a custom-built, CPU-based cluster computer designed for (among other tasks) mounting spiking-neural-network simulations. Porting and mapping aspects are detailed, while interesting insights arise as to the automatic use of the most performant backend, subject to model (that is, user-input) characteristics. Our findings pave the way for further improvements in terms, for instance, of combining multiple, heterogeneous backends for different model parts and of new simulator intelligence that can automate optimal backend selection in the future.

## Data Availability

The datasets presented in this study can be found in online repositories. The names of the repository/repositories and accession number(s) can be found in the article's Supplementary material.

## Footnotes

^1^i.e. forgoing design shortcuts that could be taken with engine-specific model descriptions.

^2^i.e., wide enough for typical computational-neuroscience projects.

^3^Refer to https://brian2.readthedocs.io/en/stable/user/running.html#scheduling.

^4^The code to reproduce this study can be found on: https://eden-simulator.org/links/paper-backends-2025-material.

^5^Due to the inter-related rapid swings in membrane voltage and channel conductance during firing, the Forward Euler method tends to better “predict” their trajectories by overshooting and typically ends up producing a more accurate firing rate than Backward Euler.

^6^For example the EDEN API could provide, instead of a full cross-referenced representation, a light-weight parser that presents so-called “chunks” of the synaptic matrix to the backend, as they are being loaded. The added complexity would then lie in the backend handling the data in such a streaming manner.
