# Supplementary material for: Decoupling model descriptions from execution: a modular paradigm for extensible neurosimulation with EDEN
Source: Front Neuroinform. 2025 Aug 7;19:1572782. doi: 10.3389/fninf.2025.1572782 (PMC12367680; doi:10.3389/fninf.2025.1572782)
Supplement: Supplementary file 1 [file Data_Sheet_1.pdf]

Supplementary Material

A IMPLEMENTING DEVICE-SPECIFIC BACKENDS

The following sections in this appendix extend the descriptions of the flexHH and SpiNNaker neural simulation platforms, and present the complete process of implementing a backend for each platform. They can be followed by SNN simulation-engine researchers and developers to integrate with the EDEN architecture and share the benefits that it offers.

A.1 Implementing the flexHH backend

Here, we resume the discussion from section 4.1.

A.1.1 Data-structure layout

During simulation, the data for neurons are laid out as three arrays:

- "Cell" array: A list of indices to where each cell's compartments lie in the "Compartment" array.
- "Compartment" array: A list of the properties intrinsic to compartments (listed above) per individual compartment, with the compartments for each cell listed in order.
- "Channel/Gate" array: A list of the HH rate formulas and their parameters, the multiplicity and activation variable of each ion-channel gate. Additionally, parameters for the ion channel (base conductance, reversal potential and whether it passes the ion species that is tracked in the ion pool), which are valid only for the last gate in the channel.

Synaptic-connectivity data are stored as a *dense matrix* of weights for each pair of neurons.

A.1.1.1 Sequential processing of ion channels and gates

Using the above described format, the gate variables of each ion channel are multiplied (also according to multiplicity) separately, to produce the ion current for each channel. Note that gate variables are updated within the pipeline, at the same time that current is evaluated based on their current value. This is in line with the Forward Euler integration scheme, but different than the modified method common in other simulators. The modified method evaluates current, updates voltage and then updates the gate variables based on the membrane voltage of the *next* timestep; this significantly improves accuracy when neurons are firing. Hence the numerical methods used impact design decisions and vice versa.

A.1.1.2 Implementing ion pools as pseudo-gates

In the flexHH encoding scheme, ion pools are modelled as follows: the gate dynamics formulae are switched to depend on the previously listed gate variable, rather than membrane voltage. Hence (as shown in the following section), within the "Channel/Gate" sequence, an element representing the *ion pool* is prepended right before that of the ion-modulated channel, and necessarily all ion channels contributing to the ion flux have to be placed *before* the ion pool's element. Still, if applicable, ion-activated ion channels could be declared in between ion sources, to evaluate the flux for an incrementally expanding set of ion channels. (Or the pipeline could be modified to clear the accumulating ion flux after processing each ion activated ion channel, if appropriate.)

A.1.2 Limitations

As hinted in the previous sections, there are some concessions in the design:

- Due to the pipeline-based processing, compartments are processed once per timestep in a strict linear sequence. This means that they must be connected in a linear chain topology for each neuron, and the Forward Euler solver is used: this limits stability to models with compartments large (that is, simplified) enough to include sufficient damping, for the simulation to remain stable. Nonetheless, this modelling constrain is in line with the Rall formulation (?) of reducing compartments over the dendritic tree with lumped equivalent zones of similar electrotonic distance and properties, into a chain topology.
- There is a rich set of allowed equations for ion channel gates, but there is no single set that covers the usual needs at this level of modelling. For one, ion channel gates of the Markov formulation are not included in that set, and the pipelined way of processing restricts that at the design level.
- There is only one type of synaptic interaction: gap junctions, on only one site per neuron that has to be at the edge of the modelled morphology, with a current-voltage curve that is fixed per implementation and has only coupling weights selectable. This was a design trade-off since the computational requirements of gap junctions are massively more than action-potential synapses, and for this very reason they have been rarely included in SNN models.

With all these limitations considered, there still is no other design for reconfigurable hardware that allows this level of multi-compartment electrophysiology modelling, with parameterised equations. The following section shows how to map NeuroML models to this architecture.

A.1.3 Mapping NeuroML models to the flexHH representation

This section shows how NeuroML models are transformed into a flexHH-native configuration, using EDEN tooling. Once the model description is provided by the `ReadNeuroML` routine, the steps to run simulations with the custom device are simple: check every part of the model to make sure it is supported, then implement the neuron populations, synaptic projections and other parameters of the simulation on the device, and – from that point on – run the simulation natively on the device.

The first of these steps (analysing the model and its parts to ensure compatibility) is the most involved one, thanks to the deep structure and diverse options that NeuroML offers for biophysically-modelled cells. Since validating support for model parts is essentially finding the matching feature of the architecture, parameter conversion can also be largely performed in-line with validation.

Each part of the SNN to be simulated is checked for flexHH compatibility and then expressed in the flexHH format, as described in the following sections and Figure S1:

1. Morphologically and biophysically detailed neuron descriptions (Section A.1.4);
2. Synaptic projections connecting the populations;
3. Experimental setup such as input probes and recorded state of neurons.

To cover for the extended needs of modellers, NeuroML allows defining mechanisms with custom dynamics through the LEMS modelling language (Cannon et al., 2014). Therefore we provide a description in LEMS for each device-supported mechanism that is not in core NeuroML already.

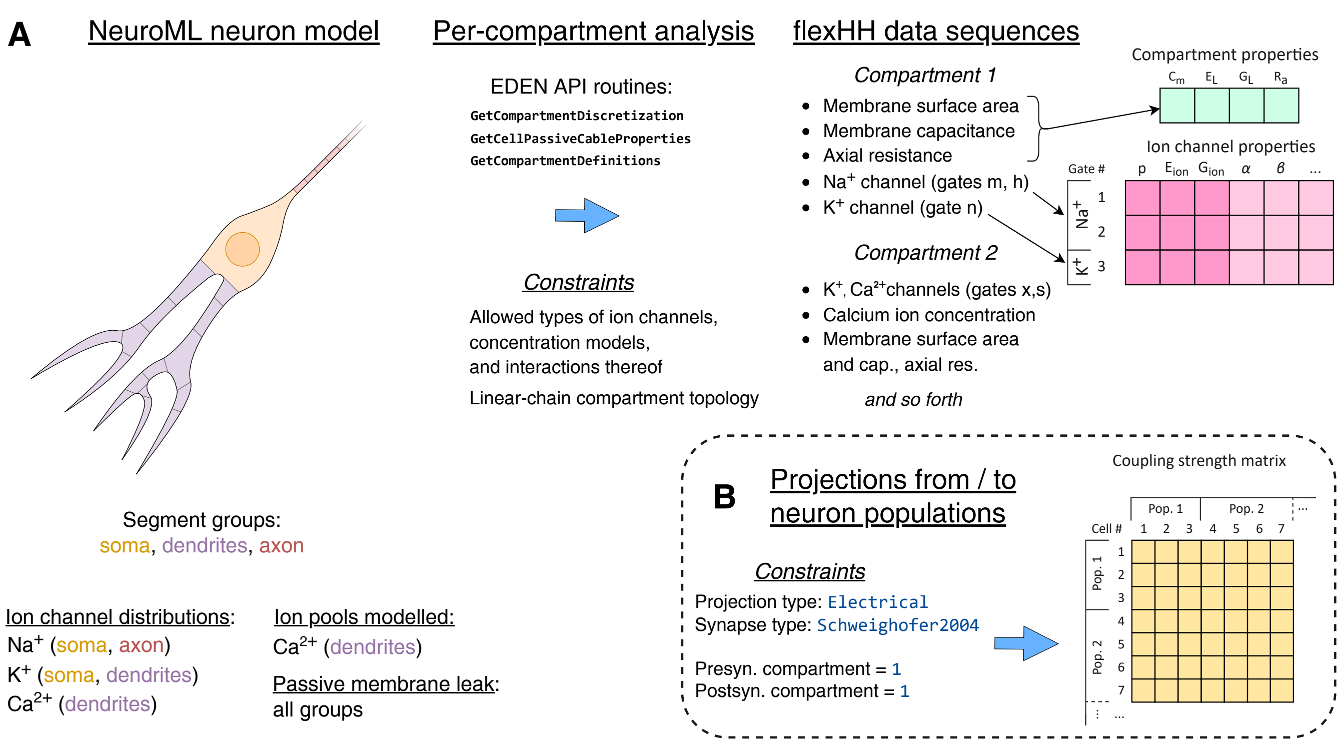

Figure S1. The NeuroML to flexHH conversion pipeline, for (a) neuron models and (b) synaptic projections.

The names of these `<ComponentType>`s are also used for detecting the device-supported mechanisms, since identification through mathematical equivalence is not tractable in the general case.

A.1.4 Cell types with spatial biophysics

The flexHH device can simulate neurons only of the NeuroML type `<cell>` which represents biophysically modelled cells; that is, abstract (also called *artificial*) cells are not supported. This is different to most of the accelerated designs (see references in Section 2.1) that were made for one or more types of point-neurons only. Since NeuroML is based on the concept of "template" cells (called "cell types") which are replicated to form a population, whereas the device's data structures hold individual data for each neuron in the model, each cell type is converted to a device-specific data structure `NeuronInfo` that gets replicated to implement populations when the model is loaded on the device.

A.1.5 Neuron structure

The first part of mapping the NeuroML models of neurons into the flexHH formulation is breaking down the neuron model into individual concrete compartments. This starts with the `GetCompartmentDiscretizationForCellType` routine, which given a NeuroML `<morphology>` generates the contiguous spans of neurite tube that each compartment represents. Then, the `GetCellPassiveCableProperties` routine extracts the compartments' biophysical cable properties in absolute terms instead of relative; that is the total area, volume, capacitance, leak and axial resistance of each compartment, instead of the prescribed specific capacitance and resistance over the extent of the tubes. There parameters are then converted to device-specific units and filled into the per-compartment records of the device-specific neuron template. Following that, the

dynamical mechanisms present on the neurites are grouped per compartment, with their properties resolved in absolute terms as well. The `GetCompartmentDefinitions` routine assembles these sets of mechanisms for each compartment, based on the neuron's spatial discretisation generated from `GetCompartmentDiscretizationForCellType`.

Before proceeding with analysing the mechanisms in each compartment, a check is made on whether the connectivity between compartments is an unbranched chain, as required by the device. The linear order of compartments also determines the layout of compartments in the device as compared to NeuroML.

A.1.6 Intra-compartment mechanisms

The mechanisms present on a compartment can be either trans-membrane ion channels transporting chemical species, or concentration models for some of these species that are being tracked, or pre/post synaptic components and stimulus probes applied externally. In this phase, information about synapses and input probes will not be considered for the device-specific data structure, as they do not affect its format (since there are already allocated parameters for each whether they are present or not). They will be converted when analysing the synaptic `<projection>` s and `<inputList>`s in the NeuroML model, as described in the following sections.

A.1.7 Ion-concentration models

Before assembling the ion channel descriptions, the ion concentration models that are present on the neuron are considered. Presently the only setup allowed by the converter is one optional population of Ca^{2+} ions, of the core NeuroML type `<fixedFactorConcentrationModel>`. There are some more options allowed by the design as explained in the previous section, that could be explored for applicable user-provided models. Due to a limitation in the specifiable dynamics rates of the device, only a steady-state concentration of zero is permitted.

As mentioned above, flexHH can keep track of the presence of one ion species per compartment (and, in theory, supersets of that ion species). Ion channels are encoded as pseudo-gate entries; these entries are inserted in the "Gate" data array between the gates for ion channels *contributing* to the ion species being tracked, and the gates which are *activated* by the concentration of that ion species as explained in Section A.1.9.1.

Hence, for each instance of concentration model being detected, its description as a device-specific, pseudo-channel data structure (see Section A.1.1.2) is filled in. This data structure can then be prepended to the descriptions for ion-activated ion channels, as explained in Section A.1.1.1, in the following.

A.1.8 Analysing and implementing ion-channel distributions

As explained in Section 4.1.2, flexHH admits a fixed base conductance per ion channel. If the conductivity is fixed or it can be directly evaluated from fixed parameters (e.g. ambient temperature), it is multiplied by the compartment's membrane surface area to yield conductance in absolute terms. If conductivity cannot be evaluated directly (e.g. depends on run-time factors), the backend rejects the SNN model. Since the device format includes parameters specifically for the static electrical leak of the membrane, the NeuroML `<ionChannel>` that fits the description, that is contains no

gates, has a linear voltage-current relationship and leaks non-specific electrical current, is detected and converted into these parameters[1].

A.1.9 Analysing and implementing gate dynamics

The analysis of the internal dynamics of the ion channels follows with the gate variables that each ion channel contains. The only gating models supported by the device are either instantaneous gating or classic HH kinetics, with independent, multiplied gates each with one open and one closed state. The dynamics equations can be expressed in terms of α, β (transition rate from closed to open and open to closed states respectively) or inf, τ (fraction of open states to converge to, and time constant of convergence) formulae; inf is also specified for instantaneous gates.

Each of these formulae that is specified in core NeuroML[2] is converted into the equivalent device-specific formula coefficients. The same, of course, is done with the formulae that are expressed through LEMS components; the difference being that they are not parsed into C++-native types by the EDEN parser API (as they are not core NeuroML types) but as instances of LEMS component types. In this case, the parameter values that apply to each component instance are converted into the device-specific coefficients. This is the case for the `FlexHH_Rate_ClippedLinearCa2` formula supported by the device, which is not a function of membrane voltage but rather a function of calcium concentration.

Another point to consider in all of the above is that, in NeuroML, gate transition rates can be scaled by a temperature-dependent speed-up factor Q_{10}. Since the ambient temperature remains fixed in simulations, this factor is evaluated for each gate and taken into account in the appropriate coefficients, for each of the aforementioned formula types.

A.1.9.1 Implementing ion-activated gates

After all gates of an ion channel have been checked for device compatibility and converted to device coefficients in channel/gate entries, they are placed in a device-specific sequence where pseudo-gates for calcium pools come before the calcium-activated ion channels. This way, the Ca^{2+} ion flux can be tracked by the pseudo-gates implementing the concentration model (see also Section A.1.1.2). Hence, the device's design cannot cover calcium-activated calcium channels, if that case appears.

A.1.10 Analysing and implementing synapses and input sources

Since flexHH offers only one option for synaptic model and input source each, mapping these is relatively simple compared to cell-model mapping:

- For each synapse in the network, check whether it is of the type that is supported by the device (e.g. Schweighofer-type non-linearity (?) for the validation and performance benchmarks in Section 5.2), and connects the first compartments of the pre- and post-synaptic cells. If the synapse is of a different type or between unsupported neuron sites, reject the model; otherwise, convert its parameters to device units, and accumulate the weight values that connect the same pair of neurons, in the connectivity matrix passed to the device.

[1] If another `<ionChannel>` fits the description, it is expressed as a device-specific ion channel without loss of generality; in theory, multiple linear leaks could be merged into a Thévenin equivalent but that is typically not the case as the effective leak conductance is determined and explicitly modelled in the first place.

[2] Namely, varieties of `HH<formula><property>` where `<formula>` can be `Exp`, `ExpLinear` or `Sigmoid` and can be `Rate`, `Time` or `Variable`; refer to the NeuroML user's guide for more details about them.

- For each input in the network, check if it is of the device-supported NeuroML type, `<pulseGenerator>`; if so, convert its delay, duration and amplitude to the in the device-native units, and assign these parameters to the relevant per-compartment data. If more than one probe targets the same compartment of the same cell, check if the timing is identical: if so, accumulate the pulse magnitude into the single resulting pulse; if not, reject the model since the device cannot simulate two separate current clamps on the same compartment.

Note: As of this writing the backend assumes one uniform neuron population in the simulated network. Supporting multiple populations is straightforward to implement: configuring the stimulus parameters and dense connectivity matrix for multiple self- and interconnected populations is a simple matter of assigning one 'global' serial number to each neuron in the network, and translating population-member references to 'global' cell numbers.

A.1.11 Model instantiation and execution

After all model parts are analysed, the device-specific description of the model data can be generated and loaded on the device: Each population's cells are instantiated by replicating the per-neuron data structure (that was constructed during cell-type analysis) for each instance of the population. Input stimulus parameters are also assigned in per-compartment data, and the synaptic weight matrix is formed by aggregating the individual synapses, as explained in the previous section.

Other simulation parameters, such as time step and total duration, are also retrieved from the `<Simulation>` description and passed to the code invoking the device. Finally, the simulation is run on the flexHH device with the user-provided model now fully translated into device-specific parameters, without further interaction with the EDEN front-end.

Models described in PyNN are set up and run through the PyNN simulation backend for SpiNNaker, called `sPyNNaker`. The following text (see also Figure S2) describes how to test a NeuroML model against the constraints mentioned above, and convert it to a PyNN-compatible description to load on `sPyNNaker`.

A.2 Implementing the SpiNNaker backend

Here, we resume the discussion from section 4.2.

A.2.1 Mapping NeuroML models to PyNN

In NeuroML, the general dynamics of SNN mechanisms (including synapses and point neurons) are expressed as abstract "component types", and different sets of parameters are expressed as different instances of these component types. Each "classic" synaptic projection designates a component instance which describes the post-synaptic mechanism; this instance is replicated to materialise each individual connection defined by projections.

The conversion process, then, consists of the following steps (see also Figure S2):

1. Verify that all cell types used are supported by PyNN.
2. Analyse synaptic projections to determine which synaptic component types are attached to which cell types, and group attached synapses per cell by model type, dynamics parameters, and excitatory or inhibitory function.

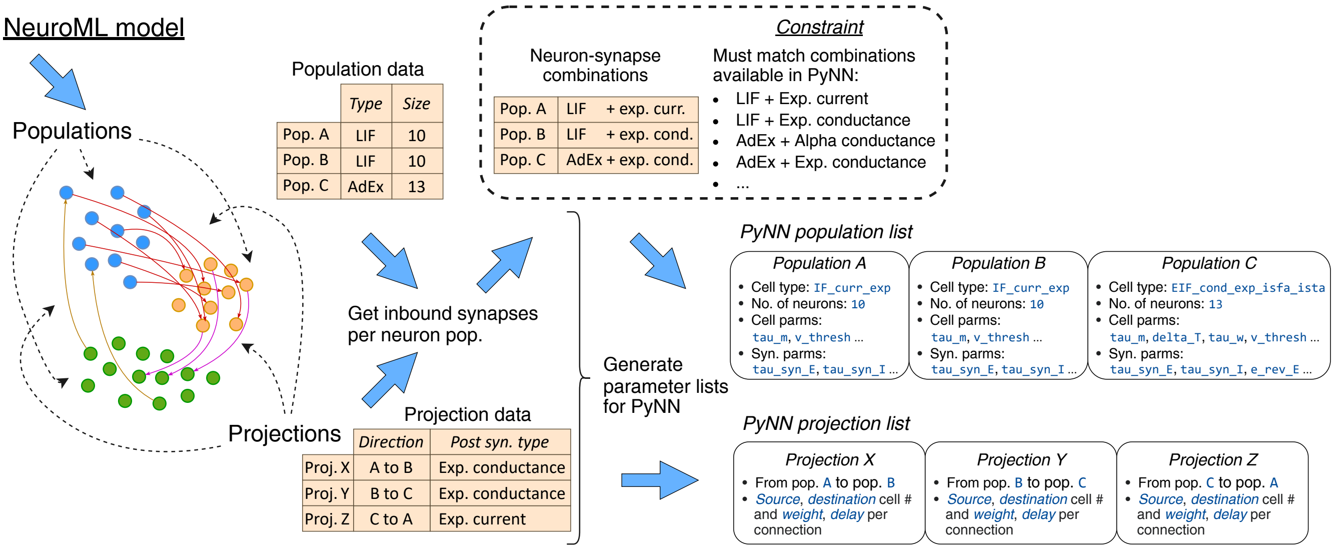

Figure S2. The NeuroML-to-PyNN conversion pipeline.

3. Match the cell types and post-synapse types to the available PyNN neuron models, and ensure that all excitatory post-synapses share the same parameters (likewise for inhibitory post-synapses), as required by PyNN.
4. Establish a list of modelled populations, each with associated size, PyNN neuron type and dynamical parameters.
5. Establish a list of modelled synaptic projections, each with its list of connections and their associated weights and propagation delays.
6. Establish a list of neuron spikes and state-variable traces to be recorded, and sundry simulation parameters, as stated in the `<Simulation>` parameters tag of the NeuroML model.
7. Instantiate the listed populations and synaptic projections between them in a PyNN backend (in this case, `sPyNNaker`), run the SNN simulation, and extract the information to be recorded for processing by the user.

A.2.2 Additional notes

Finally, it should be noted that PyNN has recently started to offer an alternative, more flexible "composed model" interface for selected backends, which accepts user-specified combinations between neuron models and post-synaptic models. This is line with the NeuroML description, and could thus allow a more direct conversion of models. However, the composed interface still offers a limited, pre-determined set of components, and the supported combinations still do not cover all neuron models in the original interface (e.g., Hodgkin-Huxley point neurons and impulse-based synapse models).

Another recent addition to the PyNN interface is some basic support for biophysics-based, multi-compartment model descriptions, much in line with the NeuroML specification; nonetheless, development of this interface is ongoing and support is limited to the very few backends which support multi-compartment modelling.

Besides the PyNN interface, SpiNNaker devices can be programmed directly at the executable-code level; in fact, the existing PyNN backend is a software application at said level, and advanced

modellers may implement custom neuron models by extending the PyNN application with model-specific C code. The NeuroML backend could thus be extended to automate this process and seamlessly support *user-provided* neuron models expressed in LEMS, by leveraging EDEN's C-code generation facilities.
